# Nitrogen and sulfur co-doped cobalt carbon catalysts for ethylbenzene oxidation with synergistically enhanced performance†

**DOI:** 10.1039/c9ra00672a

**Published:** 2019-03-25

**Authors:** Sheng Chen, Yujie Wu, Shanshan Jie, Chak Tong Au, Zhigang Liu

**Affiliations:** State Key Laboratory of Chemo/Biosensing and Chemometrics, College of Chemistry and Chemical Engineering, Hunan University Changsha 410082 China liuzhigang@hnu.edu.cn; College of Chemistry and Chemical Engineering, Hunan Institute of Engineering Xiangtan 411104 China

## Abstract

Heteroatom doping has been demonstrated to be an effective strategy for improving the performance of catalysts. In this paper, cobalt carbon catalysts co-doped with nitrogen and sulfur (N and S) were synthesized through a hydrothermal method with chelate composites involving melamine, thioglycolic acid (C_2_H_4_O_2_S), and tetrahydrate cobalt acetate (Co(OAc)_2_·4H_2_O). In addition, the selective oxidation of ethylbenzene under solvent-free conditions with molecular oxygen was used as a probe reaction to evaluate the activity of the catalysts. The optimized catalyst shows an ethylbenzene conversion of 48% with an acetophenone selectivity of 85%. Furthermore, the catalysts were systematically characterized by techniques such as TEM, SEM, XRD, Raman, and XPS. The results reveal that the species of cobalt sulfides and synergistic effects between N and S has inserted a key influence on their catalytic performance.

## Abstract 2

Co-N-S-C catalysts with rod-like structures were synthesized for the selective oxidation of ethylbenzene using O_2_ as an oxidant.

## Graphical abstract

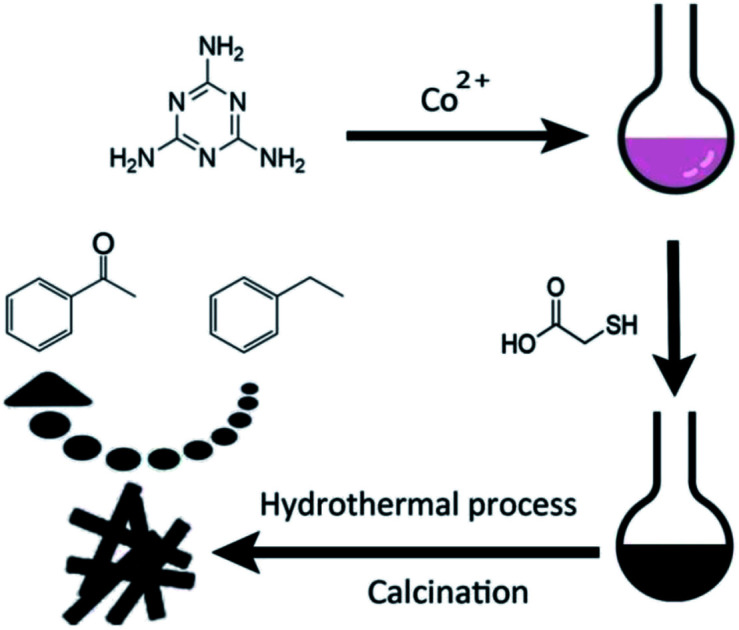

## 1. Introduction

Heterogeneous catalytic oxidation reactions are essential processes in chemical industries because the products are widely applied for the manufacture of perfumes, resins, pharmaceuticals, and so on.^1^ The selection of catalysts determines the reaction rate as well as the type and yield of products. For excellent performance, noble metals (Pd, Pt, Au, *etc.*) and strong oxidizers were used.^2^ However, the high cost and environmental limitation of these materials limits the development of this kind of catalytic system. Recently, there is an increasing interest in the use of catalysts that are based on non-noble transition metals due to their low cost and high availability. Nonetheless, the performance of these catalysts needs further improvement.^3^

Nowadays, carbon based materials, such as graphene, carbon nanotubes, activated carbon, and carbon spheres *etc.*, are essential materials in many domains.^4^ With in-depth researches, the excellent performance of carbon-based materials has been repeatedly demonstrated.^5^ It is noted that pure carbon materials without modification display poor performance in heterogeneous catalysis. Researchers combined non-noble transition metals and carbon-based materials for the formation of “non-noble transition metal carbon-based materials” (denoted herein as NMCs), in which the metal provides active sites while the carbon-based material serves as support. The goal is to prevent aggregation and loss of metal particles, thus ensuring catalytic performance superior to that of the carbon-based material. For example, iron nanoparticles enclosed in graphitic shells are known for catalytic interactions since the last century.^6^ However, despite the research conducted on NMCs, their performance is far short from industrial applications. Since 2009, Dai and coworkers reported that as a metal-free electrocatalyst for oxygen reduction reaction, nitrogen-containing carbon nanotubes worked significantly better than carbon nanotubes, in which N facilitates a four-electron pathway for the reaction.^7^ Among heteroatom dopants, N is most frequently used to enhance electron transfer ability and defect generation.^8^ Beside N, heteroatoms such as B and P can also improve the catalytic performance of carbon-based materials. In recent years, the co-doping of carbon materials with two different heteroatoms is considered promising for the enhancement of catalytic performance.^9^ For instance, mesoporous carbon materials doped with N and S are promising catalysts for water remediation where the dopants in the active phase play a key role.^10^

Traditionally, catalytic oxidation processes are conducted in liquid phase, leading to high capital cost as a result of catalyst separation for recycling.^11^ The oxidation of substrates under solvent-free conditions using O_2_ (or air) as oxidant is ideal, and efforts were put in to explore green protocols, and significant progresses have been achieved.^12^ In this article, we report N and S co-doped cobalt carbon catalysts synthesized by a hydrothermal method, using melamine as nitrogen and carbon source, C_2_H_4_O_2_S as sulfur source, and Co(OAc)_2_·4H_2_O as cobalt precursor. The catalysts were characterized by TEM, SEM, Raman, XRD, and XPS techniques. The catalytic performance of the catalysts was evaluated using the selective oxidation of ethylbenzene as model reaction.

## 2. Experimental section

### 2.1. Catalyst preparation

In the present study, the catalysts were synthesized hydrothermally. In a 100 ml round-bottom flask, 3 mmol of melamine was dissolved in 50 ml of deionized water at 100 °C to form a homogeneous solution, which was heated to reflux for 15 min after adding 1 mmol of Co(OAc)_2_. Then 2 mmol of C_2_H_4_O_2_S was added into the resulted solution, and the mixture was continuously stirred for 1 h. After that, the obtained mixture was transferred to a 75 ml stainless-steel autoclave for hydrothermal reaction at 200 °C for 18 h in a blast oven. After cooling down to room temperature, the mixture was subject to filtration and the as-obtained solid substance was washed several times with abundant amount of deionized water. The collected black precipitate was dried overnight at 80 °C in an oven. Finally, the solid was finely ground and transferred into a small quartz boat and heated to 700 °C at a heating rate of 5 °C min^−1^ in a tube furnace and then calcined at 700 °C for 90 min in a nitrogen atmosphere. The substance was well ground and is herein denoted as Co-N-S-C-700.

For comparison purposes, samples denoted as Co-N-S-C-600, Co-N-S-C-800 and Co-N-S-C-900 were prepared similarly but with calcination conducted at 600, 800, and 900 °C, respectively. For the control experiments, Co-N-C-700, Co-S-C-700 and Co-N-S-C-700 catalysts were prepared. For the synthesis of Co-N-C-700, 3 mmol of melamine, 50 ml of distilled water and 1 mmol of Co(OAc)_2_·4H_2_O were mixed and stirred for 1 h, and the resulted mixture was processed as described in the case of Co-N-S-C-700. Similarly, the Co-S-C-700 sample was prepared having melamine replaced by 3 mmol of glucose monohydrate. As for the generation of N-S-C-700, the Co-N-S-C-700 sample was treated with 50 ml of 4 M hydrochloric acid at 90 °C for 4 h.

### 2.2. Catalyst characterization

Transmission electron microscopy (TEM) was executed on a Tecnai G2 F20 S-TWIN implement operated at 120 kV and a Philips CM200 FEG implement operated at 200 kV to visualize the size and morphology of the samples and probe the effect of cobalt, nitrogen and sulfur on sample structure and morphology. Field emission scanning electronic microscopy (SEM) operated on a JSM-6700F microscope and scanning transmission electron microscopy (STEM) performed on JEM-2100F for elemental mapping were conducted for further analysis of sample morphology. X-ray diffraction (XRD) investigation was carried out on a Japan XRD-6100 diffractometer using Ni-filtered Cu Kα radiation (50 kV, 10 mA). The surface elemental compositions of samples were measured by X-ray photoelectron spectroscopy (XPS) conducted on a PHI 5000C ESCA system (PerkinElmer). Raman spectra were recorded with a 632.8 nm laser on a LabRAM Aramis micro Raman spectrometer at ambient temperature.

### 2.3. Catalytic activity test

In the present study, the oxidation of ethylbenzene with molecular oxygen (O_2_) under solvent-free conditions was used to test the catalytic performance of the prepared samples. Quintessentially, 28 mg of catalysts and 10 ml of ethylbenzene were added into a Teflon-lined autoclave, and after being well sealed, the system was heated to a designated temperature (*e.g.*, 120 °C). Then, the oxygen valve was adjusted to keep the system under 0.8 MPa of O_2_, and the reaction was maintained at 120 °C for 5 h with continuous magnetic stirring. At length, ethylbenzene conversion and product selectivity were measured by gas chromatography over an Echrom A90 instrument equipped with a HP-5 ms capillary column, employing bromobenzene (0.2 g) and *p*-dichlorobenzene (0.02 g) as internal standard.

After the oxidation of ethylbenzene, the reaction solution was centrifuged and the obtained black powder was washed with anhydrous ethanol for three times and then dried in an oven at 80 °C overnight; the as-resulted dry black powder is herein denoted as Co-N-S-C-700-*R*. The Co-N-S-C-700-*R* sample was calcined at 700 °C in nitrogen once again, and the obtained sample was denoted as r-Co-N-S-C-700-2. Accordingly, the sample after another cycle of reaction is denoted as r-Co-N-S-C-700-3. In this paper, all the characterizations for the re-activated catalyst were based on r-Co-N-S-C-700-3.

## 3. Results and discussion

### 3.1. Morphology and structure analysis

**Scheme 1:**
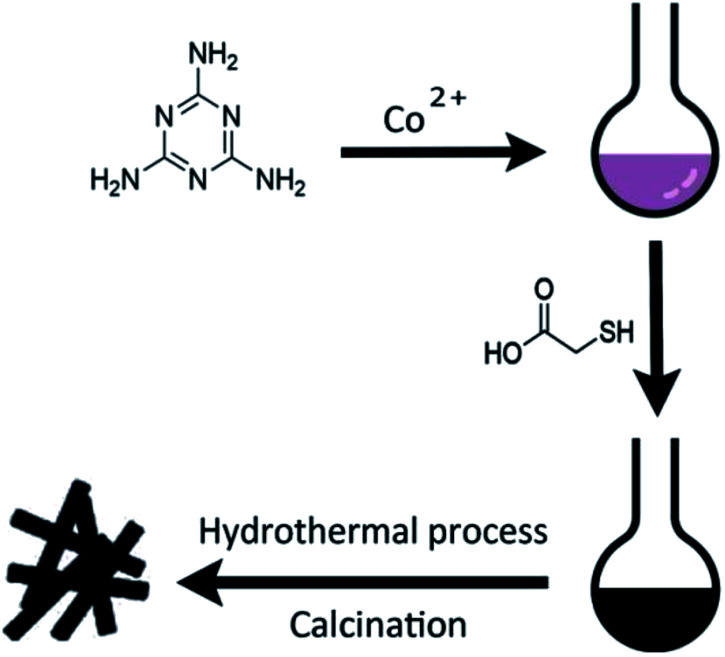
Schematic illustrations of the synthesis process of the representative Co-N-C-*X* catalysts and the catalytic reaction for ethylbenzene.

**Fig. 1:**
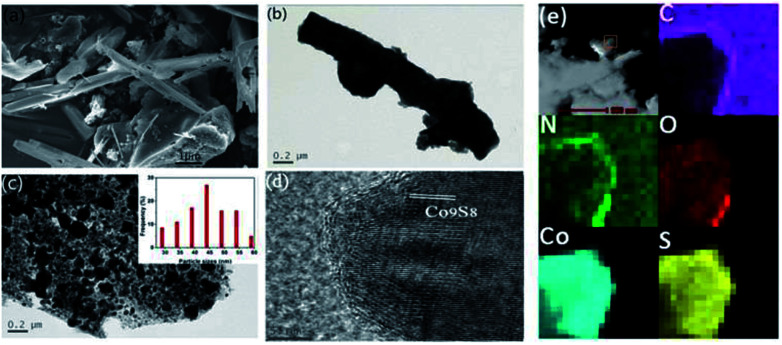
(a) SEM image of Co-N-S-C-700; TEM images of (b) Co-N-S-C-700 and (c) Co-N-C-700 (inset is the size distribution of Co-N-C-700 particles); (d) high-resolution TEM image of Co-N-S-C-700; (e) STEM image and elemental mappings of Co-N-S-C-700.

Scheme 1 illustrates the general procedure for the synthesis of the Co-N-S-C-*T* (*T* stands for the temperature adopted for catalyst calcination) catalysts. Generally speaking, during the one-pot pyrolysis of precursor molecules involving melamine, Co(OAc)_2_·4H_2_O and C_2_H_4_O_2_S, there is the gradual generation of brownish black precipitates as a result of homogeneous chelation. After hydrothermal carbonization at 200 °C and calcination at a designated temperature (600, 700, 800 or 900 °C), the targeted samples were finally obtained. Fig. 1a displays the structure and morphology of Co-N-S-C-700 as obtained in scanning electron microscopy (SEM). It is clear that a portion of the N,S co-doped cobalt carbon materials are with rod-like rather than hollow structure,^13^ and they are essentially irregular in size. It can be observed that these rod-like structures are different in length and width (Fig. S1a†), similar phenomena were reported before, indicating the effectively of this method in forming different rod structures.^14^ As depicted in the transmission electron microscopy (TEM) images of Fig. 1b, c and S1b,† the Co-N-S-C-700, Co-N-C-700 and Co-S-C-700 samples are different in morphology and structure.^15^ In Fig. 1b, a rod-like structure of Co-N-S-C-700 is displayed, and the coarse structure is estimated to be 0.36 μm in width.^14*b*,16^ The lattice spacing of particles marked in red lines in Fig. 1d measured to be 0.3 nm is corresponding to the (311) plane of Co_9_S_8_. Moreover, the elemental mapping images of Co-N-S-C-700 demonstrate the existence of C, N, O, Co and S (Fig. 1e), which indicates the successful synthesis of the catalyst. Also, there is uniform distribution of N in the Co-N-S-C-700 catalyst. As showed in Fig. 1c, Co-N-C-700 is in the form of particles with size ranging from 19 to 59 nm (inset), in a way similar to that reported by Wang *et al.*,^17^ and a small portion of the particles are with size of around 100 nm. In contrast to Co-N-C-700, the Co-S-C-700 sample has rod-like structures with little detection of particles (Fig. S1b†), indicating that the C_2_H_4_O_2_S precursor has an effect on the formation of rod-like entities. Moreover, the entities of Co-S-C-700 are closely aggregated, while those of the other samples are more dispersed, indicating the N atoms can act as anchoring sites for better dispersion of metal particles as suggested by Chen *et al.*^18^

**Fig. 2:**
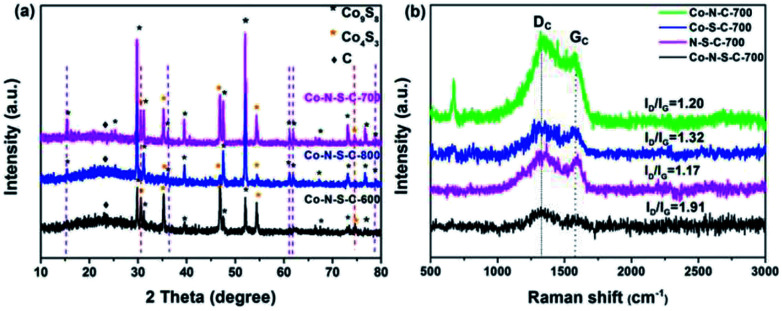
(a) XRD patterns of Co-N-S-C-600, Co-N-S-C-700, and Co-N-S-C-800; (b) Raman spectra of Co-N-C-700, Co-S-C-700, Co-N-S-C-700, and N-S-C-700.

The X-ray diffraction (XRD) patterns of the Co-N-S-C-*T* samples are depicted in Fig. 2a. The broad peak at 2*θ* = 23.3° belongs to the (002) planes of graphitic carbon. Nearly all the peaks of the samples can be assigned to crystals of cobalt sulfides. There are eight peaks that are assignable to face-centered cubic Co_9_S_8_ (JCPDS no. 19-0364). Five of the Co_9_S_8_ diffraction peaks (indicated by purple dashed lines) can be detected over Co-N-S-C-700 and Co-N-S-C-800 but not over Co-N-S-C-600. Beside the peaks of Co_9_S_8_, there are three intense diffraction peaks at 2*θ* = 35.3°, 46.8° and 54.2° attributable to the (101), (102), and (110) reflections of Co_4_S_3_ (JCPDS no. 02-1458). Two of these three peaks (marked by red dashed lines) can be detected over Co-N-S-C-600 and Co-N-S-C-700 but not over Co-N-S-C-800. It is apparent that the diffraction peaks of Co-N-S-C-700 are sharper and bigger than those of Co-N-S-C-600 and Co-N-S-C-800, indicating higher crystallinity of the former.^19^ What is more, Co-N-S-C-600 with the lowest calcination temperature is the lowest in crystallinity. There is no detection of signals ascribable to impure phases, demonstrating the high purity of samples.^20^ It is worth pointing out that there are no characteristic peaks of cobalt nitride over the samples, which could be due to the low content and/or high dispersion of nitride compounds.^21^

The Raman spectra of Co-N-C-700, Co-S-C-700, Co-N-S-C-700, and N-S-C-700 are showed in Fig. 2b. There is the obvious presence of D and G band at 1339 and 1594 cm^−1^, respectively. The former is related to lattice defects while the latter to in-plane stretching vibration of sp^2^ carbon atoms.^22^ The D band is more intense than the G band of the four samples.^23^ The *I*_D_/*I*_G_ value of Co-N-S-C-700, Co-S-C-700, Co-N-C-700, N-S-C-700 is 1.91, 1.32, 1.20, and 1.17, respectively. The remarkably high *I*_D_/*I*_G_ value of Co-N-S-C-700 suggests that the presence of Co and the co-doping of N and S promote the formation of defective sites and vacancies in the graphite lattice. In other words, the amount of structural defects in graphite increases due to the presence of transition metals and the co-doping of heteroatoms.^24^ Moreover, it was observed that the *I*_D_/*I*_G_ value decreases with the increase of calcination temperature, indicating that the degree of graphitization increases with the rise of calcination temperature (Fig. S2†).

X-ray photoelectron spectroscopy (XPS) analysis was carried out to obtain information about the compositions and chemical states of surface elements.^25^ The survey spectra indicate the presence of C, N, O, S, and Co (Fig. S3a†). The O 1s signals could be due to adsorbed water and/or oxygen species.^26^ As indicated in Table S1,† there is obvious difference in N-S-Co composition across the Co-N-S-C-*T* samples. Due to the lack of N-S-Co regularity, it is deduced that not only the relative content of cobalt and heteroatoms but also their bonding mode with the graphitic structure play an essential role in catalytic performance.

**Fig. 3:**
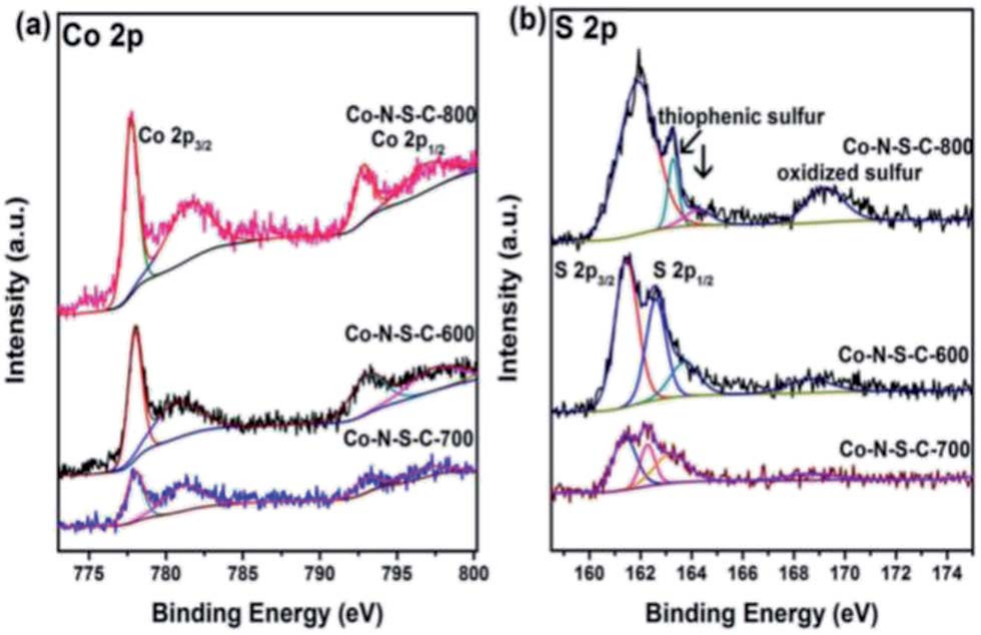
High-resolution XPS spectra of Co-N-S-C-600, Co-N-S-C-700 and Co-N-S-C-800: (a) Co 2p and (b) S 2p.

In Fig. 3a, the two sets of peaks at *ca.* 780 eV and 797 eV binding energies are Co 2p_3/2_ and Co 2p_1/2_ signals, respectively.^27^ The Co 2p_3/2_ peaks can be deconvoluted into two components at 777.9 and 781.3 eV. The former is due to metallic Co while the latter Co^3+^ and Co^2+^.^28^ Similar phenomena can be observed with the Co 2p_1/2_ peaks. It is noted that there are features of Co 2p_3/2_ satellite at *ca.* 786 eV corresponding to Co^3+^ and/or Co^2+^ species. The acquired S 2p spectra are illustrated in Fig. 3b. The S 2p spectra can be deconvoluted into four peaks at 161.4, 162.3, 163.3 and 168.7 eV, with the last one in line with that of oxidized sulfur. The two peaks at around 161.4 and 162.3 eV are S 2p_3/2_ and S 2p_1/2_ signals, respectively, of Co–S entities in Co-N-S-C-*T*. As for the S 2p peak at 163.3 eV, it is attributed to the C–S–C and C=S bonds of thiophenic S.^29^

As displayed in Fig. S3b,† the C 1s peaks at 284.4, 283.9, 285.8 and 288.9 eV can be ascribed to C–C & C=C, C–S, C–N, and C–O, respectively. The detection of C 1s signals assignable to C–N and C–S suggests the successful doping of N and S atoms into the Co-N-S-C-*T* catalysts, in consistent with the results of elemental mappings (Fig. 1e).^30^ The N 1s profiles can be deconvoluted into four peaks (Fig. S3c†) attributable to pyridinic N (398.4 eV), pyrrolic N (399.2 eV), graphitic N (401.0 eV) and oxidized N (403.9 eV).^31^ The N 1s signals of Co-N-S-C-600 and Co-N-S-C-700 are nearly undetectable whereas that of Co-N-S-C-800 is relatively strong. It is apparent that when the catalyst calcination temperature is 800 °C, there is the presence of a high proportion of pyridinic N which may serve as coordination sites with cobalt ions, leading to high dispersion of active sites apt for catalytic reaction.^32^

### 3.2. Catalytic performance analysis

Aerobic oxidation of ethylbenzene is an environmentally-friendly process widely used for the manufacture of chemical intermediates.^33^ Thus, the selective oxidation of ethylbenzene under solvent-free conditions was chosen as probe reaction to evaluate the performance of the as-prepared catalysts. The intrinsic nature of the catalysts in terms of catalyst recovery and product separation was also considered.^34^ The catalytic performance of Co-N-S-C-*T* catalysts was tested and showed in Table 1. In the absence of a catalyst, ethylbenzene conversion is low and selectivity to acetophenone is 79% (Table 1, entry 1). On the basis of the results in Table 1, it is obvious that the temperature adopted for catalyst calcination has a significant influence on catalytic activity. Among the Co-N-S-C-*T* catalysts, Co-N-S-C-700 is the highest in ethylbenzene conversion (48%), followed by Co-N-S-C-800 (25%), Co-N-S-C-600 (17%) and Co-N-S-C-900 (14%) (Table 1, entries 2–5), indicating that 700 °C is the most suitable temperature for catalyst calcination. It is apparent that there are overt differences in ethylbenzene conversion but the variation in product distribution is less significant. The sharp variation of ethylbenzene conversion reiterates that a proper calcination temperature is a key factor in the synthesis efficient catalyst.

**Table 1:** Catalytic activity of different catalysts for ethylbenzene oxidation with O_2_^a^

Entry	Catalyst	Conv.^b^ (%)	Sel.^c^ (%)		
			AP	PA	BA
1	—	6	79	19	2
2	Co-N-S-C-600	17	82	17	1
3	Co-N-S-C-700	48	85	14	1
4	Co-N-S-C-800	25	83	16	1
5	Co-N-S-C-900	14	81	18	1
6	Co-N-C-700	11	82	17	1
7	Co-S-C-700	13	84	14	2
8	N-S-C-700	14	86	13	1

a Reaction conditions: ethylbenzene (10 ml), catalyst (28 mg), O_2_ (0.8 MPa), 120 °C and 5 h.

b Conversion of ethylbenzene.

c Selectivity to acetophenone (AP), phenethyl alcohol (PA) and benzaldehyde (BA).

In order to elucidate the effect of dopant on catalytic performance, a number of control experiments were conducted under the optimized conditions. It has been generally accepted that the doping of heteroatoms into the carbon materials may result in the changes in charge and spin densities of adjacent carbon atoms. Moreover, the kind of doped heteroatoms, the doping level and type of bonds formed between dopants and carbon atoms can also exert an key influence on the changes in charge and spin density.^35^ As we all know, N atoms have a stronger electronegativity and S atoms have a weaker electronegativity, compared with C atoms. Hereafter, the changes in charge and spin density of carbon atoms can be tailored by controlling the doping of N and S, which may enhance the activity of the as-prepared catalysts *via* the synergistic effect N and S. Thus, the role of nitrogen and sulfur atoms in catalytic activity was investigated over the Co-N-S-C-700, Co-S-C-700 and Co-N-C-700 catalysts. It can be seen that Co-N-S-C-700 is superior to the other two in catalytic activity (Table 1, entries 3, 6 and 7), suggesting the existence of synergistic effect between N and S for the better performance of the former. As shown in Table S2,† the effect of dopant on catalytic performance can also be observed in other reported works. To study the influence of cobalt nanoparticles on catalytic activity, Co-N-S-C-700 has treated in 4 M hydrochloric acid at 90 °C for 4 h. Over the as-obtained N-S-C-700A sample, ethylbenzene conversion (14%) is low, indicating the need of cobalt for high catalytic performance. According to the Raman spectra, Co-N-S-C-700 has the highest *I*_D_/*I*_G_ value that corresponds to the presence of defects, which should be a combined effect of nitrogen, sulfur and cobalt. It is hence deduced that nitrogen, sulfur, and cobalt species are essential for the carbon-based system.

**Fig. 4:**
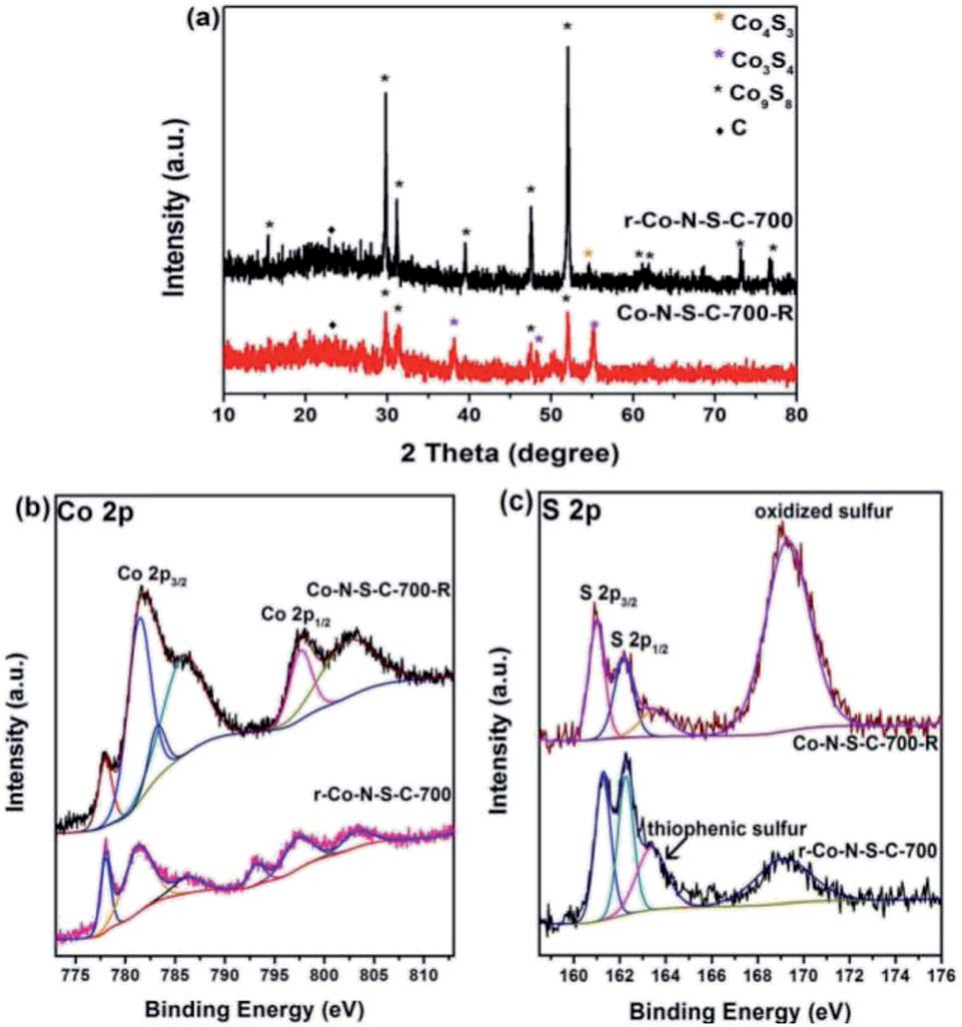
(a) XRD patterns of r-Co-N-S-C-700 and Co-N-S-C-700-*R*; high-resolution XPS spectra of (b) Co 2p and (c) S 2p for Co-S-N-C-700-*R* (used) and r-Co-S-N-C-700 (re-activated).

It is undeniable that catalyst stability is a key factor in the judging of catalyst efficiency. Unfortunately, the Co-N-S-C-700 catalyst shows a sharp decrease in catalytic activity in the second reaction cycle (Table S3,† entry 1). Nonetheless, the used catalyst can be re-activated by calcination at 700 °C for 90 min in a nitrogen atmosphere. It can be seen from entries 2, 3 of Table S3† that r-Co-S-N-C-700-2 and r-Co-S-N-C-700-3 have ethylbenzene conversion of 49% and 53%, respectively. To explain the high catalytic activity of the re-activated catalyst, we had the r-Co-S-N-C-700 and Co-S-N-C-700-*R* samples characterized. From the TEM images of r-Co-N-S-C-700, it is clear that after the ethylbenzene oxidation reaction and being subjected to calcination, the catalyst breaks down from the rod-like structure to particles without any specific morphology (Fig. S1c†). As revealed in the XRD patterns of Fig. 4a, beside the ten peaks of Fig. 2a, a weak diffraction peak at 54.2° is detected over r-Co-N-S-C-700, which is attributable to the (110) planes of Co_4_S_3_. Three new peaks at 38°, 50.3°, and 55° ascribable to the (400), (511), and (440) planes of Co_3_S_4_ (JCPDS no. 42-1448) are observed over the Co-N-S-C-700-*R* sample; which are not detected over the other catalysts in the present study. What is more, the peaks at 73.3° and 76.7° attributed to Co_9_S_8_ are absent in the case of Co-N-S-C-700-*R*. It is clear that in comparison to Co-N-S-C-700-*R*, r-Co-N-S-C-700 shows Co_9_S_8_ peaks that are significantly higher in intensity. It is observed that there is no detection of Co_4_S_3_ peaks over Co-N-S-C-700-*R* but rather those of Co_3_S_4_, suggesting that Co_4_S_3_ can be generated during calcination of Co-N-S-C-700-*R* at 700 °C in nitrogen. It can hence be stated that the presence of Co_4_S_3_ and Co_9_S_8_ has a positive effect on catalytic performance. Also, by comparing the XRD patterns of high-performance Co-N-S-C-700 with the low-performance Co-N-S-C-600 and Co-N-S-C-800, the importance of Co_4_S_3_ and Co_9_S_8_ in the catalytic reaction can also be defined.

In the Co 2p spectra of r-Co-N-S-C-700 and Co-N-S-C-700-*R*, there are strong and weak shake-up satellite features at around 793.3 eV ascribable to spin–orbit components as previous literature reported before (Fig. 4b),^30^ confirming the presence of surface Co–O components. Compared to r-Co-S-N-C-700, Co-N-S-C-700-*R* shows peaks assignable to S–O_*x*_–S and C–O bonds of higher intensity (Fig. 4c and S3b†), illustrating the enrichment of inactive oxidative groups during the ethylbenzene oxidation reaction in the expanse of the active groups as reflected in the high oxygen content of the Co-N-S-C-700-*R* sample (Table S1†). On the contrary, the content of active groups such as thiophenic S is relatively higher in the re-activated r-Co-S-N-C-700 sample. In addition, it is noted that there is the presence of fluorine in Co-N-S-C-700-*R*, which might be resulted from the polytetrafluoroethylene lining (Table S1†). The sharp decrease of catalytic activity could be ascribed to fluorine, which verified in previous work of our group.^36^ According to previously studies, the oxidation of ethylbenzene with O_2_ is a free radical reaction.^11,36^ Being strongly electron-withdrawing, fluorine is a winner in capturing the unpaired electrons of free radicals.

## 4. Conclusion

We have successfully synthesized the N,S co-doped cobalt carbon catalysts by hydrothermal carbonization process with precursor molecules involving melamine, C_2_H_4_O_2_S, and Co(OAc)_2_·4H_2_O. Among the Co-N-S-C-*T* samples, Co-N-S-C-700 exhibits the best catalytic performance for the selective oxidation of ethylbenzene using O_2_ as oxidant under solvent-free conditions. Combining the results of controlled experiments and those of TEM, SEM, XRD, Raman, and XPS analyses, it is deduced that the outstanding performance of Co-N-S-C-700 is ascribable to Co_9_S_8_ and Co_4_S_3_ active sites as well as the synergistic effect between N and S.

## Conflicts of interest

There are no conflicts to declare.

## Supplementary Material

RA-009-C9RA00672A-s001

## References

[cit1] Westerhaus F. A., Jagadeesh R. V., Wienhöfer G., Pohl M. M., Radnik J., Surkus A. E., Rabeah J., Junge K., Junge H., Nielsen M., Brückner A., Beller M. (2013). Nat. Chem..

[cit2] Jang Y., Kim S., Jun S. W., Kim B. H., Hwang S., Song I. K., Kim B. M., Hyeon T. (2011). Chem. Commun..

[cit3] Zhang F., Zhao C., Chen S., Li H., Yang H., Zhang X. M. (2017). J. Catal..

[cit4] Chen J. Y., Zhang H. M., Liu P., Li Y. B., Li G. Y., An T. C., Zhao H. J. (2015). Carbon.

[cit5] Titirici M. M., White R. J., Brun N., Budarin V. L., Su D. S., Monte F., Clarkd J. H., MacLachlan M. J. (2015). Chem. Soc. Rev..

[cit6] Cui X., Li Y., Bachmann S., Scalone M., Surkus A. E., Junge K., Topf C., Beller M. (2015). J. Am. Chem. Soc..

[cit7] Gong K., Du F., Xia Z., Durstock M., Dai L. (2009). Science.

[cit8] Yang C., Chen Y., Zhao S., Zhu R., Liu Z. (2016). RSC Adv..

[cit9] Sasan K., Kong A., Wang Y., Chengyu M., Zhai Q., Feng P. (2015). J. Phys. Chem. C.

[cit10] Roldán L., Marco Y., García-Bordejé E. (2016). Microporous Mesoporous Mater..

[cit11] Lin X., Nie Z., Zhang L., Mei S., Chen Y., Zhang B., Zhu R., Liu Z. (2017). Green Chem..

[cit12] Su Q., Yao X., Cheng W., Zhang S. (2017). Green Chem..

[cit13] Jagadeesh R. V., Murugesan K., Alshammari A. S., Neumann H., Pohl M. M., Radnik J., Beller M. (2017). Science.

[cit14] Wan H., Ji X., Jiang J., Yu J., Miao L., Zhang L., Bie S., Chen H., Ruan Y. (2013). J. Power Sources.

[cit15] Han W., Zhao Y., Dong F., Zhang G., Lu G., Tang Z. (2017). Microporous Mesoporous Mater..

[cit16] Taylor K. M., Rieter W. J., Lin W. (2008). J. Am. Chem. Soc..

[cit17] Wang H., Sun X., Liu Z., Lie Z. (2014). Nanoscale.

[cit18] Chen Y., Fu L., Liu Z. (2015). Chem. Commun..

[cit19] Zou S., Burke M. S., Kast M. G., Fan J., Danilovic N., Boettche S. W. (2015). Chem. Mater..

[cit20] Jiang J., Zhang X. I., Sun P., Zhang L. (2011). J. Phys. Chem. C.

[cit21] Wang W., Liu S., Nie L., Cheng B., Yu J. (2011). J. Phys. Chem. C.

[cit22] Gong J., Lin H., Antonietti M., Yuan J. (2016). J. Mater. Chem. A.

[cit23] Liang J., Jiao Y., Jaroniec M., Qiao S. Z. (2012). Angew. Chem., Int. Ed..

[cit24] Srinivas G., Zhu Y., Piner R., Skipper N., Ellerby M., Ruoff R. (2010). Carbon.

[cit25] Cai F. P., Xiao G. M. (2016). Catal. Sci. Technol..

[cit26] Liu N., Yin L., Wang C., Zhang L., Lun N., Xiang D., Qi Y., Gao R. (2010). Carbon.

[cit27] Chen F., Kreyenschulte C., Radnik J., Lund H., Surkus A. E., Junge K., Beller M. (2017). ACS Catal..

[cit28] Fu L., Chen Y., Liu Z. (2015). J. Mol. Catal. A: Chem..

[cit29] Yang L., Cai Z., Hao L., Xing Z., Dai Y., Xu X., Pan S., Duan Y., Zou J. (2017). ACS Appl. Mater. Interfaces.

[cit30] Gao Y., Zhao H., Chen D., Chen C., Ciucci F. (2015). Carbon.

[cit31] Fu L., Lu Y., Liu Z., Zhu R. (2016). Chin. J. Catal..

[cit32] Chen Y., Jie S., Yang C., Liu Z. (2017). Appl. Surf. Sci..

[cit33] Min B. K., Friend C. M. (2007). Chem. Rev..

[cit34] Xu J., Chen T., Shang J. K., Long K. Z., Li Y. X. (2015). Microporous Mesoporous Mater..

[cit35] Gao Y., Hu G., Zhong J., Shi Z., Zhu Y., Su D. S., Wang J., Bao X., Ma D. (2013). Angew. Chem., Int. Ed..

[cit36] Lin X., Jie S. S., Liu Z. G. (2018). Mol. Catal..

