# Supplementary material for: Nitrogen and sulfur co-doped cobalt carbon catalysts for ethylbenzene oxidation with synergistically enhanced performance
Source: RSC Adv. 2019 Mar 25;9(17):9462–7. doi: 10.1039/c9ra00672a (PMC9062161; doi:10.1039/c9ra00672a)
Supplement: RA-009-C9RA00672A-s001 [file RA-009-C9RA00672A-s001.pdf]

Electronic Supplementary Material (ESI) for RSC Advances.
This journal is © The Royal Society of Chemistry 2019

Supporting Information

Nitrogen and sulfur co-doped cobalt carbon catalysts for ethylbenzene oxidation with synergistically enhanced performance

Sheng Chen,[a] Yujie Wu,[a] Shanshan Jie,[a] Chak Tong Au,[b] and Zhigang Liu[a,*]

[a] State Key Laboratory of Chemo/Biosensing and Chemometrics, College of Chemistry and Chemical Engineering, Hunan University, Changsha 410082, China

[b] College of Chemistry and Chemical Engineering, Hunan Institute of Engineering, Xiangtan, 411104, China

Corresponding author

E-mail: liuzhigang@hnu.edu.cn (Z.G. Liu)

Materials

Melamine, $Co(OAc)_2{\cdot}4H_2O$, $C_2H_4O_2S$, glucose monohydrate ($C_6H_{12}O_6{\cdot}H_2O$), bromobenzene, 1,4-dichlorobenzene, and anhydrous ethanol were from commercial sources and were used without any further purification.

Results and discussion

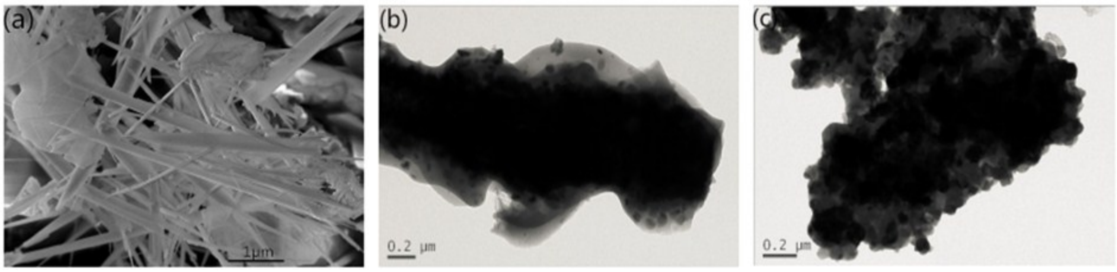

Figure S1. SEM image of (a) Co-N-S-C-700; TEM images of (b) Co-S-C-700 and (c) r-Co-N-S-C-700 (re-activated)

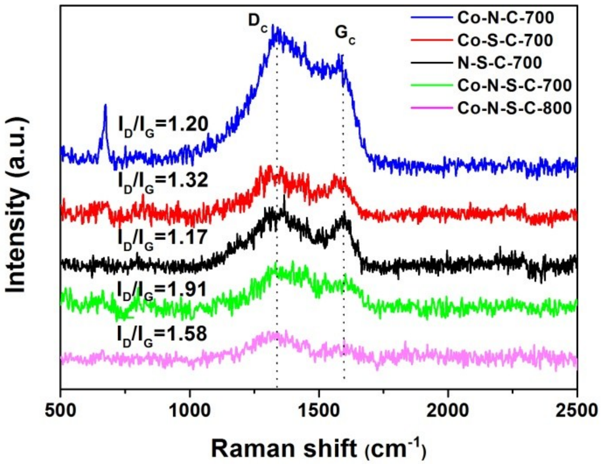

Figure S2. Raman spectra of Co-N-C-700, Co-S-C-700, N-S-C-700, Co-N-S-C-700, and Co-N-S-C-800 samples

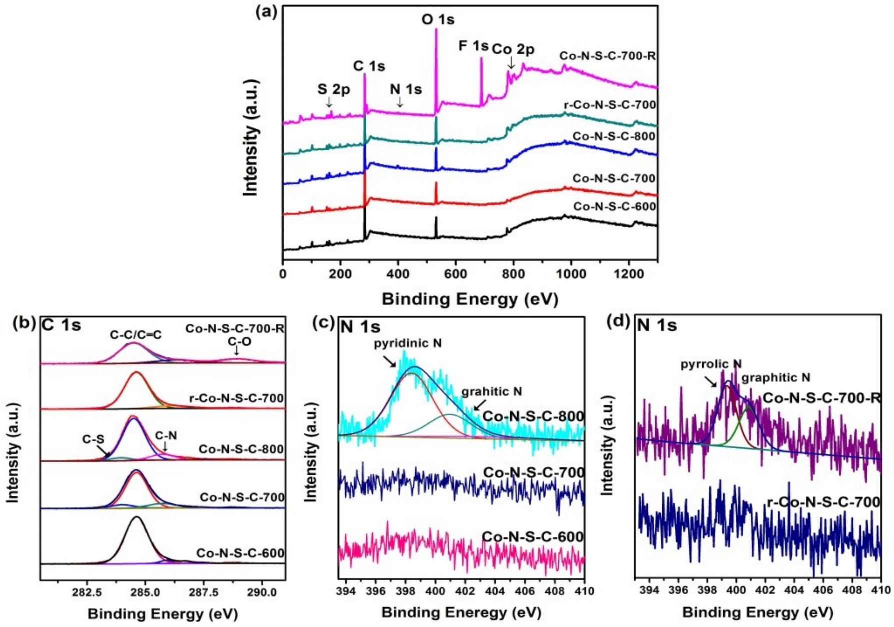

Figure S3. (a) XPS survey spectra of various Co-N-S-C-T samples; (b) High-resolution C 1s spectra of Co-N-S-C-T; High-resolution N 1s spectra of (c) Co-N-S-C-T, and (d) Co-N-S-C-700-R and r-Co-N-S-C-700 samples

Table S1. Chemical composition of different catalysts based on XPS data

Sample	Content (at%)					
	C	N	O	S	Co	F
Co-N-S-C-600	73.78	1.99	12.76	9.40	2.09	-
Co-N-S-C-700	74.81	2.63	11.81	9.43	1.32	-
Co-N-S-C-800	74.85	3.19	9.35	10.66	1.95	-
r-Co-N-S-C-700	72.77	1.17	13.24	9.29	3.52	-
Co-N-S-C-700-R	42.45	1.15	28.40	11.59	6.41	10.00

Table S2. The comparison of the work and the recently reported work

Catalysts	Dopants	Conv.(%)[a]	Select.(%)[b]	Cond.[c]	Ref.
Co-N-S-C-700	N, S	48	85	0.8 MPa O_2, 120 °C, 5h	This work
Co@GCNs-800	N	68.1	93.2	0.8 MPa O_2, 120 °C, 5h	[1]
S-CoNC	N, S	62	91	0.8 MPa O_2, 120 °C, 5h	[2]
Co-N-C-Phen	N	40	75	0.8 MPa O_2, 120 °C, 5h	[3]
Co_3O_4@GNC	N	65.8	72.6	20 bar air, 140 °C, 4h	[4]
CoNC-10	N	22	75	0.8 MPa O_2, 120 °C, 5h	[5]

[a] Conversion of ethylbenzene; [b] Selectivity to acetophenone; [c] Condition of reaction.

Table S3. Recycling performance of Co-N-S-C-700 in oxidation of ethylbenzene with O_2[a]

Entry	Catalyst	Conv.(%)[b]	Sel.(%)[c]		
			AP	PA	BA
1	Co-N-S-C-700-R	8	88	10	2
2	r-Co-N-S-C-700-2	49	86	13	1
3	r-Co-N-S-C-700-3	52	87	13	0

[a] Reaction conditions: ethylbenzene (10 ml), catalyst (28 mg), O_2 (0.8 MPa), 120 °C and 5 h.[b] Conversion of ethylbenzene. [c] Selectivity to acetophenone (AP), phenethyl alcohol (PA) and benzaldehyde (BA).

[1] X. Lin, Z. Z. Nie, L. Y. Zhang, S. C. Mei, Y. Chen, B. S. Zhang, R. L. Zhu and Z. G. Liu, Green Chem., 2017, 19,2164-2173.

[2] X. Lin, S, S, Jie and Z. G. Liu, Mol. Catal, 2018, 455, 143–149.

[3] S. S. Jie, C. Q. Yang, Y. Chen and Z. G. Liu, Mol. Catal, 2018, 458, 1–8.

[4] S. Pendem, R. Singuru, C. Sarkar, B. Joseph. J. Lee, D. B. Shinde, Z. P. Lai and J. Mondal, Appl. Nano. Mater. (ACS). 2018, 1, 4836−4851.

[5] Y. Chen, L. Fu, Z. Liu, Chem. Commun. 2015, 51, 16637–16640.
